# Thermal Transport Investigation in Magneto-Radiative GO-MoS_2_/H_2_O-C_2_H_6_O_2_ Hybrid Nanofluid Subject to Cattaneo–Christov Model

**DOI:** 10.3390/molecules25112592

**Published:** 2020-06-02

**Authors:** Syed Tauseef Mohyud-Din, Adnan, Umar Khan, Naveed Ahmed, Ilyas Khan, T. Abdeljawad, Kottakkaran Sooppy Nisar

**Affiliations:** 1 University of Multan, Multan 60000, Pakistan; syedtauseefs@hotmail.com; 2 Department of Mathematics, Mohi-ud-Din Islamic University, Nerian Sharif AJ&K 12080, Pakistan; adnan_abbasi89@yahoo.com; 3 Department of Mathematics and Statistics, Hazara University, Mansehra 21120, Pakistan; umar_jadoon4@yahoo.com; 4 Department of Mathematics, Faculty of Sciences, HITEC University, Taxila Cantt 47070, Pakistan; nidojan@gmail.com; 5 Department of Mathematics, College of Science Al-Zulfi, Majmaah University, Al-Majmaah 11952, Saudi Arabia; 6 Department of Mathematics and General Sciences, Prince Sultan University, Riyadh 11 586, Saudi Arabia; 7 Department of Medical Research, China Medical University, Taichung 40402, Taiwan; 8 Department of Computer Science and Information Engineering, Asia University, Taichung 40402, Taiwan; 9 Department of Mathematics, College of Arts and Sciences, Prince Sattam bin Abdulaziz University, Wadi, Aldawaser 11991, Saudi Arabia; n.sooppy@psau.edu.sa

**Keywords:** thermal radiations, thermal transport, Cattaneo–Christov heat flux model, magnetic field, GO-MoS_2_/C_2_H_6_O_2_-H_2_O hybrid nanofluid, thermophysical characteristics

## Abstract

Currently, thermal investigation in hybrid colloidal liquids is noteworthy. It has applications in medical sciences, drug delivery, computer chips, electronics, the paint industry, mechanical engineering and to perceive the cancer cell in human body and many more. Therefore, the study is carried out for 3D magnetized hybrid nanofluid by plugging the novel Cattaneo–Christov model and thermal radiations. The dimensionless version of the model is successfully handled via an analytical technique. From the reported analysis, it is examined that Graphene Oxide-molybdenum disulfide/C_2_H_6_O_2_-H_2_O has better heat transport characteristics and is therefore reliable for industrial and technological purposes. The temperature of Graphene Oxide GO-molybdenum disulfide/C_2_H_6_O_2_-H_2_O enhances in the presence of thermal relaxation parameter and radiative effects. Also, it is noted that rotational velocity of the hybrid nanofluid rises for stronger magnetic parameter effects. Moreover, prevailed behavior of thermal conductivity of GO-molybdenum disulfide/C_2_H_6_O_2_-H_2_O is detected which shows that hybrid nanofluids are a better conductor as compared to that of a regular nanofluid.

## 1. Introduction

Thermal transportation and analysis of thermophysical characteristics of the nanomaterials by incorporating the concept of Lorentz forces, thermal radiations and Cattaneo–Christov constitutive model is very popular among the researchers and engineers due to their numerous applications.

The inspection of nanofluid heat transfer by considering Cattaneo–Christov heat flux bounded between radiative plates was reported in [1]. For fascinating behavior of the velocity, they incorporated the effects of Lorentz forces in energy equation. Further, they detected that by incorporating the Cattaneo–Christov model, the temperature rises. In 2018, Mahmood et al. [2] examined the entropy analysis in the boundary layer flow of Casson nanofluid. For thermal transport improvement, they used the Cattaneo–Christov model in the energy equation. They pointed that Cu-H_2_O is better for thermal transport in comparison with TiO_2_-H_2_O colloidal mixture. They highlighted the negative variations in the nanofluid motion and thermal behavior enhanced for non-Newtonian parameter. Intensifications in the heat transport for nanoliquid between parallel disks are presented in [3]. The disks are capable to stretching and for novelty of the model, they plugged the Cattaneo–Christov heat flux in the energy equation. They implemented the Duan–Rach technique, which is a modified version of the Adomians Decomposition technique for examining the impacts of plugged physical quantities in the flow behavior.

The flow characteristics of Carreau fluid by plugging the Cattaneo–Christov model over a slendering surface were examined in [4]. The decrement in thermal transport and thermal boundary layer is inspected by incorporating Cattaneo–Christov constitutive model. The influence of Cattaneo–Christov constitutive equation on thermal transport of a Carbon nanotubes (CNT)-based colloidal mixture is investigated in [5]. They carried the numerical computations for the model and considered multiple slip conditions. The numerical inspection of entropy analysis for magnetized squeezed flow of colloidal mixture by plugging the Cattaneo–Christov constitutive relation was reported in [6]. The analysis of hybrid aluminum alloys (AA7072 and AA7075) diluted in methanol is discussed in [7]. Also, they incorporated Lorentz forces effects in the momentum constitutive relation and detected that heat transfer rate is larger for AA7072-AA7075/methanol hybrid colloidal mixture.

The study of magnetized Casson nanofluid by plugging the activation energy and thermal radiation in the chemically reacting nonlinearly stretchable surface described in [8]. The influence of multiple quantities on the entropy generation was the major concerns of their analysis. Recently, Rasool and Zhang [9] pointed the Darcy–Forchheimer flow of the colloidal mixture inspired by Cattaneo–Christov theory. They carried the flow nonlinearly stretchable surface and pointed the influence of numerous preeminent parameters in the flow behavior. The impacts of various pertinent flow quantities on the flow and thermal behavior of TiO_2_-CuO/H_2_O hybrid colloidal solution over a wedge under the different wedge conditions are described in [10].

In 2019, Shah et al. [11] explored heat and mass transportation in the flow of micropolar nanofluid between radiative rotating sheets. They incorporated the concept of ohmic heating the energy relation and found fascinating behavior of the thermal field. Inspection of entropy generation in porous channel for the hybrid nanomaterial based colloidal fluid described by Das et al. [12]. For hybrid nanomaterial they used the mixture of Cu-Al_2_O_3_ and H_2_O is used as host fluid. They encountered the fascinating behavior of numerous parameters on irreversibility and thermal transportation. Recently, the detection of Ag-Fe_3_O_4_-H_2_O hybrid nanoliquid was reported in [13]. The problem was modeled between Riga geometry and Fourier law and chemical reaction were induced in the energy and concentration constitutive relations, respectively. The entropy analysis for radiative hybrid nanofluid and the behavior of induced Lorentz forces was reported in [14]. 3D squeezed flow regimes for hybrid nanoliquid was examined in [15]. A comparative analysis for the hybrid and conventional nanoliquids by inducing the forced convection characteristics was described in [16]. The analysis of thermal transport for tiny particles and molecular diameters and squeezed flow by incorporating the microcantilever sensor was described in [17] and [18], respectively. 3D flow of colloidal suspension between circular plates perceived in [19]. The was study conducted in the presence of microorganisms and ferro nanofluid. Another significant analysis related to microorganisms by considering Lorentz forces was described in [20].

Thermal transport by inducing the radiative Cattaneo–Christov constitutive model in magnetized GO-MoS_2_/H_2_O-C_2_H_6_O_2_ has not been examined thus far. Hybrid nanofluids have enriched thermal transport characteristics and is very fruitful for the industrial, engineering and technological applications. The hybrid problem is modeled in the radiative rotating channel with a lower porous end. The hybrid problem is explored in terms of thermal transport, shear stresses and the impacts of volume friction ϕ on the hybrid nanofluid effective characteristics. Finally, outcomes of the analysis incorporated in the conclusion.

## 2. Results and Discussion

### 2.1. GO-Molybdenum Disulfide/H_2_O-C_2_H_6_O_2_ Velocity Behaior

#### 2.1.1. The Variations in F(η) and F′(η) for A * and Ω *

**Figure 1:**
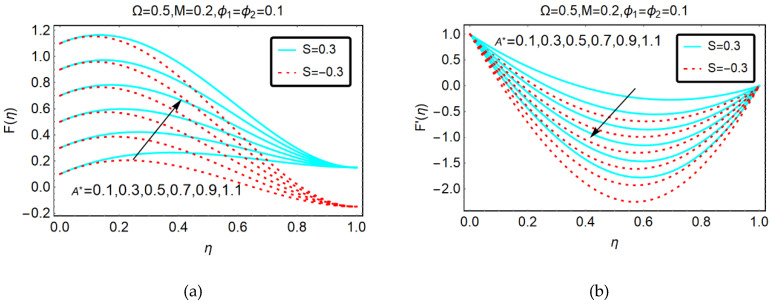
Behavior of (**a**) F(η) and (**b**) F′(η) against A*.

The behavior of GO-molybdenum disulfide/H_2_O-C_2_H_6_O_2_ velocities F(η) and F′(η) against the suction effects A* are shown in Figure 1. It is worthy to mention that lower end of the channel is static and downwards and apart movement of the upper end is due to S > 0 and S < 0, respectively. Moreover, the colloidal suspension between the plates due to downwards movement of the upper plate.

It is perceived that the GO-molybdenum disulfide/H_2_O-C_2_H_6_O_2_ flow rapidly for alike and away movement of the plates by increasing the suction effects. Physically, when the upper accelerates in a downwards direction, then the nanofluid is squeezed and the plate exerts a force on the fluid particles. Due to the downwards movement of the plate, the suction effects prevail and consequently, the velocity rises. For the plate to move apart, slow acceleration in the hybrid nanofluid is detected. Physically, when the plate moves in an away direction, then the flowing area increases and more hybrid nanofluid particles drag to fill in the free space; therefore, the velocity enhances quite slowly.

Figure 1b portrays the reversal behavior of F′(η) against suction effects. It is examined that GO-molybdenum disulfide/H_2_O-C_2_H_6_O_2_ moves slowly for stronger squeezing effects and back flow is pointed out for near the lower end. Physically, the suction of the fluid provides free space due to which the back flow produces. Therefore, the velocity F′(η) drops abruptly. 

**Figure 2:**
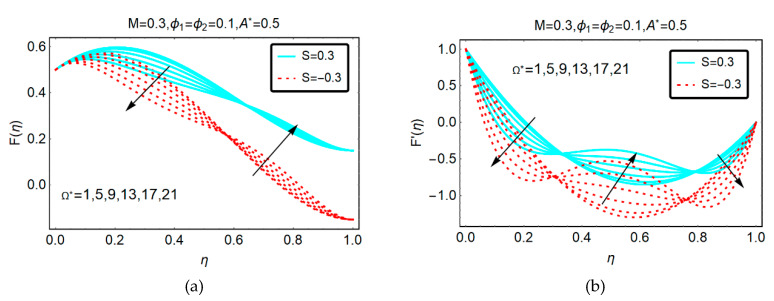
Behavior of (**a**) F(η) and (**b**) F′(η) against $\Omega^{*}.$

The velocity of GO-molybdenum disulfide/H_2_O-C_2_H_6_O_2_ due to rotation of the plates is shown in Figure 2. The velocity shows dual variations by altering the rotational parameter. It is noticed that the squeezing effects prevailed for downwards S > 0 movement of the plate as comparative to apart movement S < 0. Furthermore, fascinating trends in the velocity F′(η) against rotational effects are shown in Figure 2b.

#### 2.1.2. Behavior of F(η) and F′(η) for M

**Figure 3:**
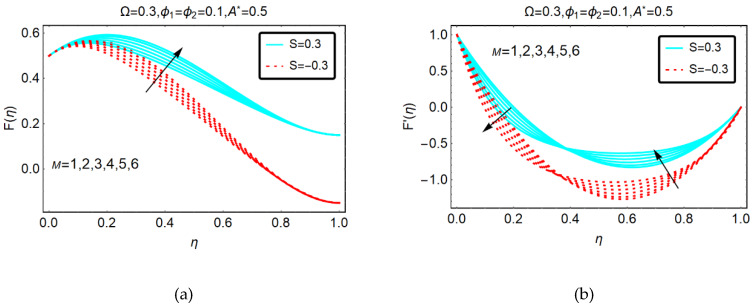
Behavior of (**a**) F(η) and (**b**) F′(η) against M.

The velocities F(η) and F′(η) against imposed magnetic field are highlighted in Figure 3. It is observed that the movement of GO-molybdenum disulfide/H_2_O-C_2_H_6_O_2_ is almost negligible near the channel boundaries. Physically, this behavior of the velocity is due to the suction effects and imposed dimensionless magnetic parameter. On other side, F′(η) is showing fascinating variations due to high magnetic parameter. it is perceived that F′(η) shows dual behavior for stronger magnetic effects.

**Figure 4:**
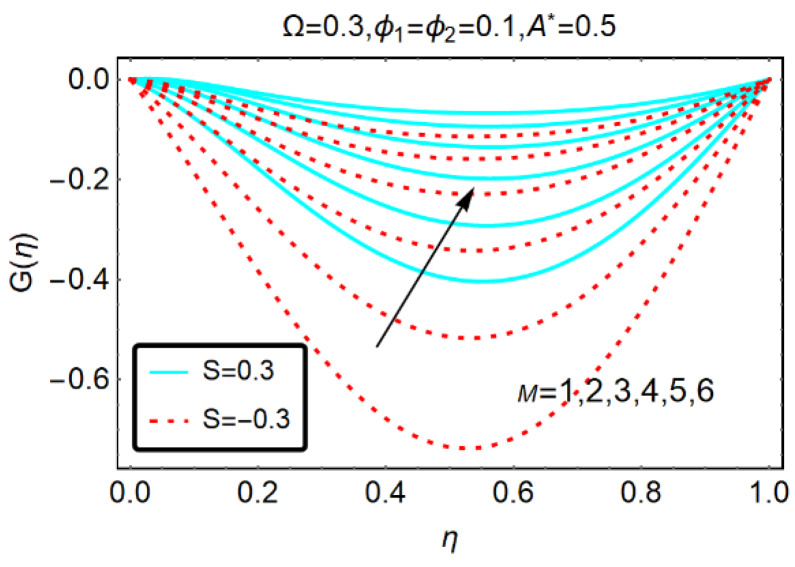
Behavior of G(η) for M.

The effects of the imposed dimensionless magnetic number on the rotational velocity G(η) are decorated in Figure 4. It is pointed out that in the middle region, a back flow is produced. Physically, the suction effects from the lower plate prevail and the imposed M resists the motion. Therefore, in the middle of the channel, more back flow trends are perceived. Furthermore, when the upper plate accelerates in a backward direction, these effects are very rapid.

### 2.2. GO-Molybdenum Disulfide/H_2_O-C_2_H_6_O_2_ Thermal Behavior

#### Behavior of Multiple Parameters on β(η)

**Figure 5:**
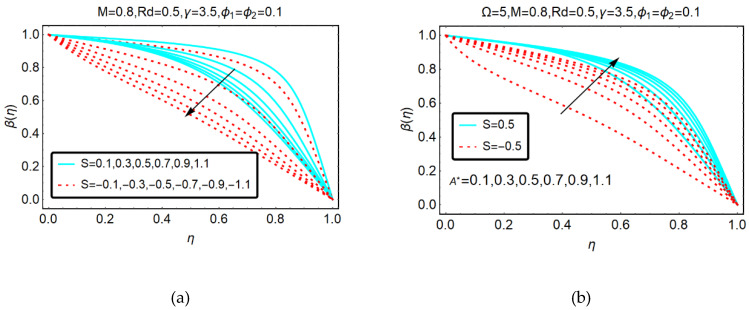
Behavior of β(η) for (**a**) S and (**b**) A*.

The temperature effects for alike and apart movement of the upper plate at constant suction are shown in Figure 5a. It can be seen that for the apart movement of plate, the temperature β(η) declines abruptly. Physically, the apart movement of the plate creates free space, due to which the velocity of the hybrid nanofluid declines. Due to drops in the velocity, the collision between the fluid particles decreases and rapid decrement in the temperature β(η) is detected. On the other side, when the upper plate accelerates downwards, then the collision between the fluid particles increases which produces energy between the fluid particles; consequently, the decrement in the temperature becomes slow.

Figure 5b shows the behavior of hybrid nanofluid temperature against multiple values of the suction parameter. The increasing behavior of the temperature is perceived for a higher suction of the fluid. It is reported that when the upper sheet moves apart from the lower sheet, then the increment in the temperature is slow. Physically, the apart movement of the upper sheet creates free space between the plates and more particles rapidly drag to fill the gap. Therefore, collision between the fluid particles increases and the temperature significantly enhances.

**Figure 6:**
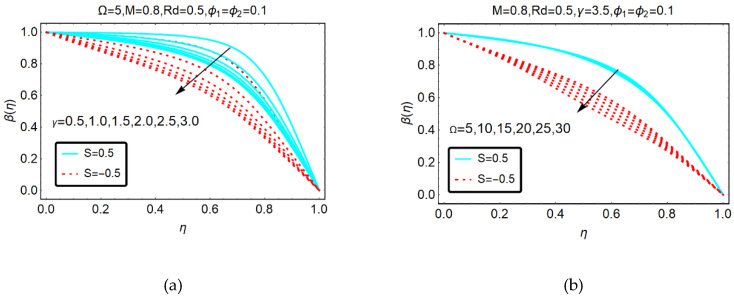
Behavior of β(η) for (**a**) γ and (**b**) Ω.

**Figure 7:**
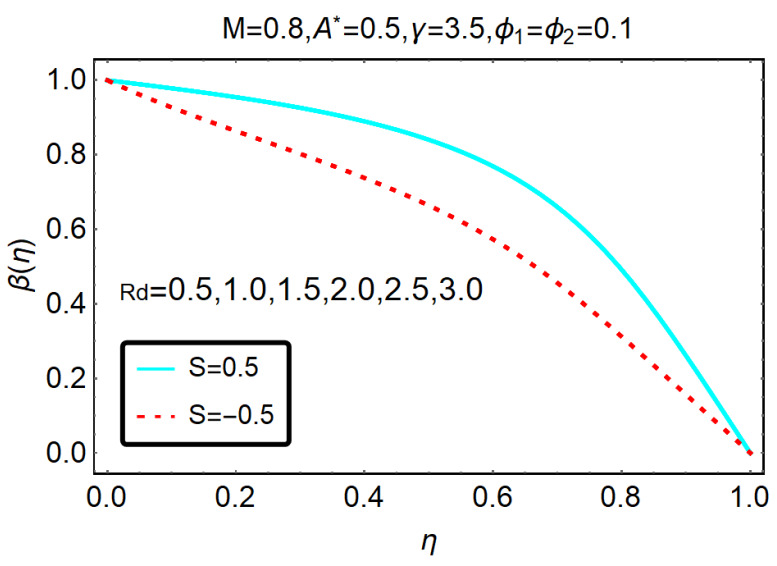
Behavior of β(η) for Rd.

The trends of the GO-molybdenum disulfide/H_2_O-C_2_H_6_O_2_ temperature for thermal relaxation γ, rotational Ω parameters and thermal radiations Rd are shown in Figure 6 and Figure 7, respectively. It is perceived that β(η) drops for the aforementioned physical parameters. However, for the thermal relaxation parameter, these effects prevailed.

### 2.3. Skin Friction and Nanofluid Contour Pattern

**Figure 8:**
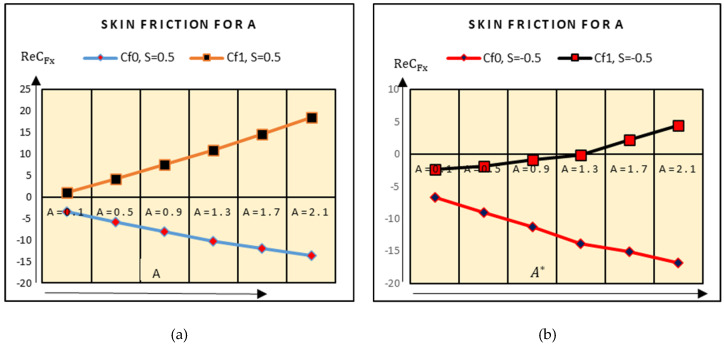
Shear stresses against suction (**a**) S = 0.5 and (**b**) S = −0.5.

This subsection deals the analysis of shear stresses at the upper and lower plate against multiple physical parameters. Figure 8 highlights the behavior of shear stresses for varying suction parameter A^*^. It is noticed that the shear stress trends increase at the upper plate for downwards movement. Physically, when the upper plate accelerates in a downward direction, then more fluid particles become adjacent; therefore, shear stresses increase. Similarly, for apart movement of the plate, the increasing behavior of the shear stresses is quite slow. Physically, the fluid particles separated from the upper plate for away movement, which leads to slow increment in the shear stress trends.

**Figure 9:**
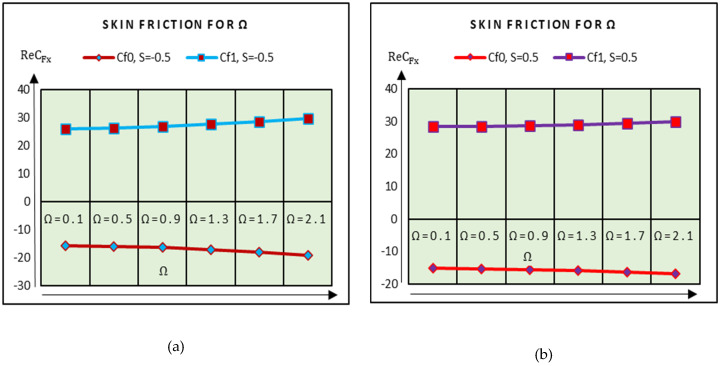
Shear stresses against Ω (**a**) S = −0.5 and (**b**) S = 0.5.

**Figure 10:**
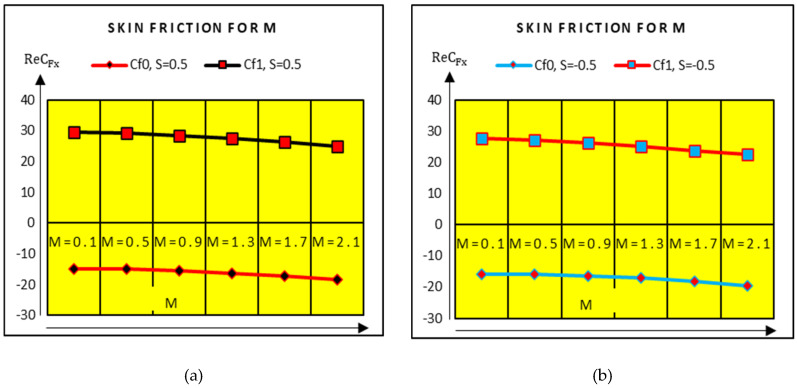
Shear stresses against Ω (**a**) S = 0.5 and (**b**) S = −0.5.

The shear stresses due to rotation of the plates are shown in Figure 9. It is examined that the shear stresses at the upper plate increase very slowly and drop at the lower plate. Therefore, the effects of rotational parameter on the shear stresses are minimal. In Figure 10, the shear stresses drop slowly at the upper and lower plates for stronger magnetic effects. At the upper plate, rapid decrement in the shear stresses is examined.

**Figure 11:**
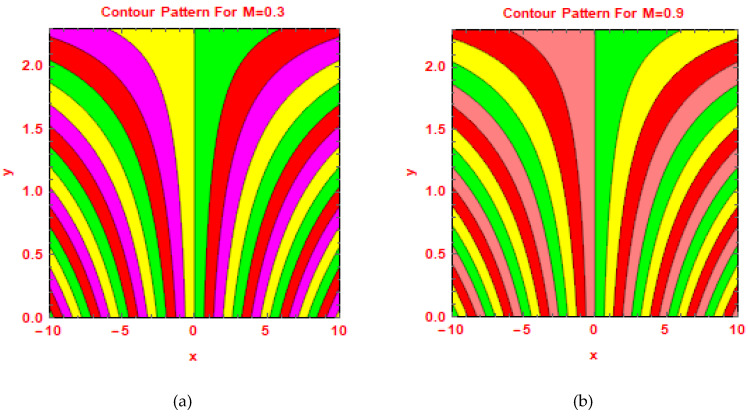
Contours for (**a**) M=0.3 and (**b**) M = 0.9.

**Figure 12:**
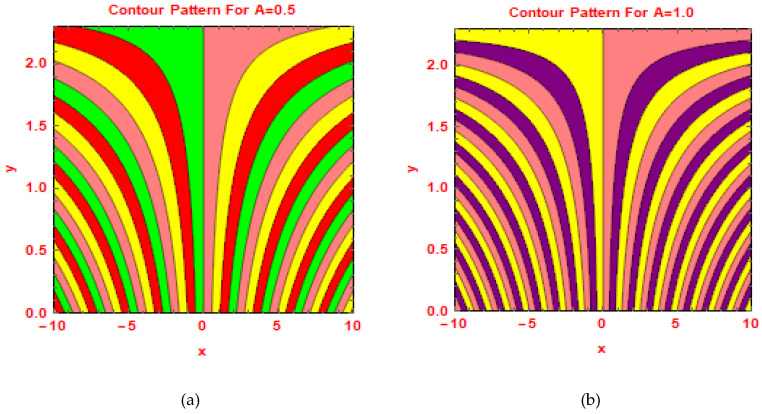
Contours for (**a**) A = 0.5 and (**b**) A = 1.0.

In Figure 11 and Figure 12, the behavior of streamlines is shown via contour plot approach for preeminent parameters.

### 2.4. The Effects of ϕ on Thermophysical Characteristics

**Figure 13:**
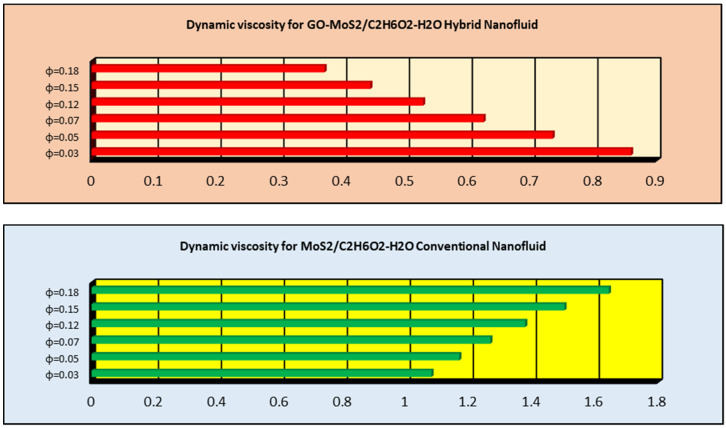
Dynamic viscosity for GO-molybdenum disulfide/H_2_O-C_2_H_6_O_2_ and MoS_2_/H_2_O-C_2_H_6_O_2_.

**Figure 14:**
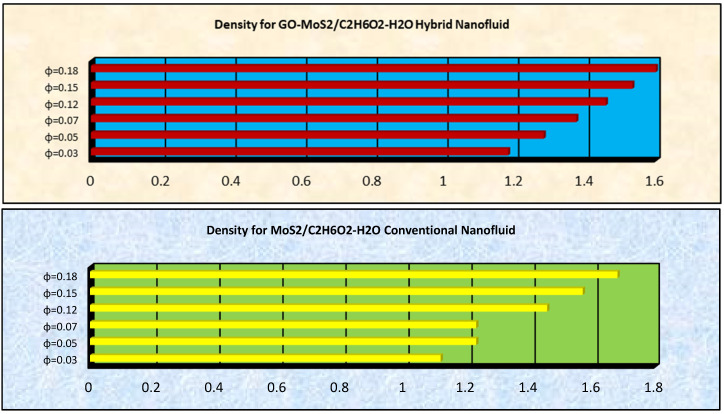
Density for GO-molybdenum disulfide/H_2_O-C_2_H_6_O_2_ and MoS_2_/H_2_O-C_2_H_6_O_2_.

**Figure 15:**
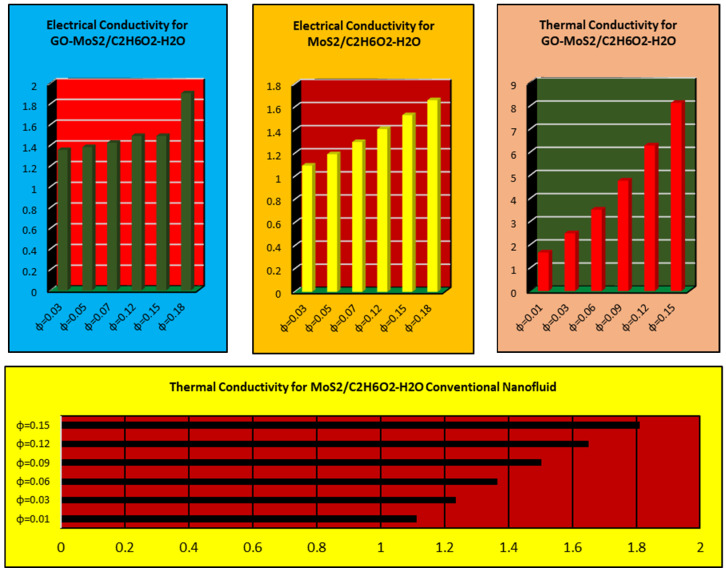
Electrical and thermal conductivities for GO-molybdenum disulfide/H_2_O-C_2_H_6_O_2_ and MoS_2_/H_2_O-C_2_H_6_O_2_.

The effects of ϕ on thermophysical characteristics of GO-molybdenum disulfide/H_2_O-C_2_H_6_O_2_ and MoS_2_/H_2_O-C_2_H_6_O_2_ are presented in this subsection. These are shown in Figure 13, Figure 14 and Figure 15. It is examined that the dynamic viscosity of MoS_2_/H_2_O-C_2_H_6_O_2_ increases for higher ϕ. Similarly, the densities of GO-molybdenum disulfide/H_2_O-C_2_H_6_O_2_ and MoS_2_/H_2_O-C_2_H_6_O_2_ increases for high volume friction nanoparticles, which significantly alters the velocity characteristics of the nanofluids. Moreover, it is explored that the thermal conductivity of GO-molybdenum disulfide/H_2_O-C_2_H_6_O_2_ prevailed, which is significant for thermal transport properties.

### 2.5. Reliablity of the Study

Table 1 presents the comparative analysis of the study with existing scientific literature. The presented analysis is in better agreement with literature results, which shows the reliability of the results and implemented mathematical technique.

## 3. Materials and Methods

### 3.1. Geometry and Statement

#### 3.1.1. Host Liquid and Nanomaterial

The 3D squeezed hybrid problem is taken in the rotating geometry in which the hybrid base liquid is H_2_O-C_2_H_6_O_2_. The hybrid nanomaterial is Go-MoS_2_. The hybrid tiny particles and host liquid are thermally in equilibrium and there is no slip between them.

#### 3.1.2. Assumptions

The following assumptions are made for the analysis: 

The hybrid liquid is viscous and incompressible.

The influence of Lorentz forces and Cattaneo–Christov constitutive relation are induced in an unsteady radiative constitutive model.

The lower plate is immovable and positioned at y = 0.

The upper end of the channel is positioned at h = (ν (1 − ct)/a)^0.5^ and is function of t.

The rotating velocity of the hybrid liquid and channel is Ω = ω_j_ × (1 − ct)^−^^1^.

The squeezed velocity of hybrid nanoliquid at the lower end is −V_o_/(1 − ct). 

U_w_ = ax (1 − ct)^−^^1^ is stretching velocity of lower end.

The strength of plugged magnetic field is B_0_ (1 − ct)^−^^0.5^.

**Figure 16:**
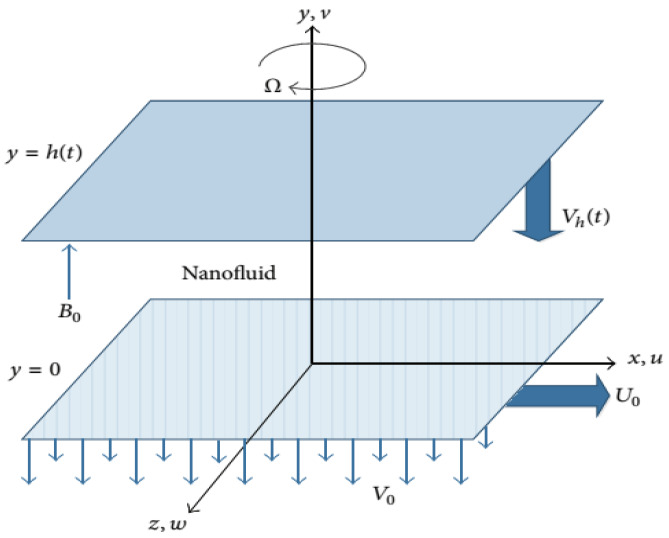
Hybrid model flow scenario.

Figure 16 illustrates the flow geometry for GO-MoS_2_/H_2_O-C_2_H_6_O_2_.

### 3.2. Governing Colloidal Model

The constitutive model for molybdenum disulfide (MoS_2_) and graphene oxide (GO) in 50:50 hybrid host liquid H_2_O-C_2_H_6_O_2_ in the occurrence of Cattaneo–Christov heat and thermal radiations described as follows [22]:

(1) $$\frac{\partial u}{\partial x}+\frac{\partial v}{\partial y}+\frac{\partial w}{\partial z}=0,\;$$

(2) $$\Xi_{1}^{*}\left(\frac{\partial u}{\partial t}+u\frac{\partial u}{\partial x}+v\frac{\partial u}{\partial y}+2\omega_{0}{\left(1-ct\right)}^{-1}w\right)=-\frac{\partial p}{\partial x}+\Xi_{2}^{*}\left(\frac{\partial^{2}u}{\partial x^{2}}+\frac{\partial^{2}u}{\partial y^{2}}\right)-\frac{\Xi_{3}^{*}B_{0}^{2}}{\left(1-ct\right)}u,\;$$

(3) $$\Xi_{1}^{*}\left(\frac{\partial v}{\partial t}+u\frac{\partial v}{\partial x}+v\frac{\partial v}{\partial y}\right)=-\frac{\partial p}{\partial y}+\Xi_{2}^{*}\left(\frac{\partial^{2}v}{\partial x^{2}}+\frac{\partial^{2}v}{\partial y^{2}}\right),$$

(4) $$\Xi_{1}^{*}\left(\frac{\partial w}{\partial t}+u\frac{\partial w}{\partial x}+v\frac{\partial w}{\partial y}-2w_{0}{\left(1-ct\right)}^{-1}u\right)=\Xi_{2}^{*}\left(\frac{\partial^{2}w}{\partial x^{2}}+\frac{\partial^{2}w}{\partial y^{2}}\right)-\frac{\Xi_{3}^{*}B_{0}^{2}}{\left(1-ct\right)}w,\;$$

(5) $$\begin{array}{l} \left(\frac{\partial T}{\partial t}+u\frac{\partial T}{\partial x}+v\frac{\partial T}{\partial y}\right)+\tau_{0}^{*}(\frac{\partial T^{2}}{\partial t^{2}}+u\frac{\partial u}{\partial x}\frac{\partial T}{\partial x}+v\frac{\partial T}{\partial y}\frac{\partial v}{\partial y}+u\frac{\partial T}{\partial y}\frac{\partial v}{\partial x}+v\frac{\partial T}{\partial x}\frac{\partial u}{\partial y}+2vu\frac{\partial^{2}T}{\partial x\partial y}+u^{2}\;\frac{\partial T^{2}}{\partial x^{2}}+\\ v^{2}\;\frac{\partial T^{2}}{\partial y^{2}}+\frac{\partial T}{\partial x}\;\frac{\partial u}{\partial t}+\frac{\partial T}{\partial y}\frac{\partial v}{\partial t}+2\;u\;\frac{\partial^{2}T}{\partial x\partial t}+2\;v\;\frac{\partial^{2}T}{\partial y\partial t})=\frac{\Xi_{4}^{*}}{\Xi_{5}^{*}}\left(\frac{\partial^{2}T}{\partial x^{2}}+\frac{\partial^{2}T}{\partial y^{2}}\right)+\frac{16\sigma^{*}T_{\infty }^{3}}{3k^{*}\Xi_{5}^{*}}\frac{\partial^{2}T}{\partial y^{2}},\; \end{array}$$

The effective characteristics of the hybrid nanoliquid in the constitutive hybrid model specified in Equations (1)–(5) are denoted by Ξ_i_* (i = 1,2…,5). The mathematical expressions for these models are as under [23]. Table 2 shows effective models for Graphene Oxide (GO) and molybdenum-disulfide based hybrid nanoliquids while, various shapes of tiny particles are decorated in Table 3.

Where, m shows the shape factor of the hybrid tiny particles. The shapes for different values of m embedded in Table 3. Thermophysical attributes of the nanomaterials and hybrid host liquid are described in Table 4.

The flow geometry is adjusted under the following conditions:

(6) $$\begin{array}{c} u=U_{w}=ax{\left(1-ct\right)}^{-1}\\ v=-V_{0}{\left(1-ct\right)}^{-1}\\ w=0\\ T=T_{w} \end{array}\}at\;y=0,\;\left(\mathrm{lower}\;\mathrm{permeable}\;\mathrm{fixed}\;\mathrm{plate}\right),\;$$

And,

(7) $$\begin{array}{c} u=0\\ v=V_{h}=-\frac{c}{2}{\left(\frac{v}{a\left(1-ct\right)}\right)}^{0.5}\\ w=0\\ T=T_{h} \end{array}\}at\;y=h\left(t\right),\;\left(\mathrm{at}\;\mathrm{upper}\;\mathrm{plate}\;\mathrm{moveable}\;\mathrm{plate}\right).\;$$

The specific invertible transformations that support the particular hybrid model defined as [22]:

(8) $$\begin{array}{c} u=U_{w}F^{\prime }\left(\frac{y}{h\left(t\right)}\right)\\ v=\sqrt{\frac{a\upsilon }{a\left(1-ct\right)}\;}\;F\left(\frac{y}{h\left(t\right)}\right)\\ w=U_{w}G\left(\frac{y}{h\left(t\right)}\right)\\ \beta \left(\frac{y}{h\left(t\right)}\right)=\frac{T-T_{w}}{T_{w}-T_{h}}\\ \eta =\frac{y}{h\left(t\right)} \end{array}\}\mathrm{invertible}\;\mathrm{transformations}.\;$$

By plugging the invertible transformations and partial derivatives into the constitutive hybrid model, the following dimensionless version is obtained.

### 3.3. GO-Molybdenum Disulfide/H_2_O-C_2_H_6_O_2_ Colloidal Model

(9) $$\begin{array}{c} F^{\prime \prime \prime }-\left(\left(1-\phi_{2}\right)\left(\left(1-\phi_{1}\right)+\phi_{1}\frac{\rho_{s1}}{\rho_{f}}\right)+\phi_{2}\rho_{s2}\right){\left(1-\phi_{1}\right)}^{2.5}{\left(1-\phi_{2}\right)}^{2.5}\left(3F^{\prime }F^{\prime \prime }-FF^{\prime \prime \prime }+\frac{S}{2}\left(3F^{\prime \prime }+\eta F^{\prime \prime \prime }\right)+2\Omega G^{\prime }\right)\;\\ -{\left(1-\phi_{1}\right)}^{2.5}{\left(1-\phi_{2}\right)}^{2.5}\left(1+\frac{3\phi \left(\phi_{1}\sigma_{1}+\phi_{2}\sigma_{2}-\sigma_{bf}\left(\phi_{1}+\phi_{2}\right)\right)}{\left(\phi_{1}\sigma_{1}+\phi_{2}\sigma_{2}+2\phi \sigma_{bf}\right)-\sigma_{bf}\phi \left(\left(\phi_{1}\sigma_{1}+\phi_{2}\sigma_{2}\right)-\sigma_{bf}\left(\phi_{1}+\phi_{2}\right)\right)}\right)M^{2}F^{\prime \prime }=0 \end{array}$$

(10) $$\begin{array}{c} G^{\prime \prime }+\left(\left(1-\phi_{2}\right)\left(\left(1-\phi_{1}\right)+\phi_{1}\frac{\rho_{s1}}{\rho_{f}}\right)+\phi_{2}\rho_{s2}\right){\left(1-\phi_{1}\right)}^{2.5}{\left(1-\phi_{2}\right)}^{2.5}\left(FG^{\prime }-F^{\prime }G+2\Omega F^{\prime }-S\left(G+\frac{\eta }{2}G^{\prime }\right)\right)\\ -{\left(1-\phi_{1}\right)}^{2.5}{\left(1-\phi_{2}\right)}^{2.5}\left(1+\frac{3\phi \left(\phi_{1}\sigma_{1}+\phi_{2}\sigma_{2}-\sigma_{bf}\left(\phi_{1}+\phi_{2}\right)\right)}{\left(\phi_{1}\sigma_{1}+\phi_{2}\sigma_{2}+2\phi \sigma_{bf}\right)-\sigma_{bf}\phi \left(\left(\phi_{1}\sigma_{1}+\phi_{2}\sigma_{2}\right)-\sigma_{bf}\left(\phi_{1}+\phi_{2}\right)\right)}\right)M^{2}G=0 \end{array}$$

(11) $$\begin{array}{c} \left(1+\frac{Rd}{\left(\frac{k_{s2}+\left(m-1\right)k_{bf}-\left(m-1\right)\left(k_{bf}-k_{s2}\right)\phi_{2}}{k_{s2}+k_{bf}\left(m-1\right)+\left(k_{bf}-k_{s2}\right)\phi_{2}}\right)}\right)\beta^{\prime \prime }-\\ \frac{\mathrm{Pr}\left(\left(1-\phi_{2}\right)\left(\left(1-\phi_{1}\right)+\phi_{1}\frac{{\left(\rho c_{p}\right)}_{s1}}{{\left(\rho c_{p}\right)}_{f}}\right)+\phi_{2}{\left(\rho c_{p}\right)}_{s2}\right)\left(\begin{array}{c} F\beta^{\prime }\\ -\frac{S}{2}\eta \beta^{\prime } \end{array}+\gamma \left(\frac{F^{\prime }\beta^{\prime }S\eta }{2}-\frac{3S^{2}\eta \beta^{\prime }}{4}-\frac{S^{2}\eta^{2}\beta^{\prime \prime }}{4}+S\eta \beta^{\prime \prime }+\frac{3SF\beta^{\prime }}{2}\begin{array}{c} --FF^{'\beta^{\prime }}\\ -F^{2}\beta^{\prime \prime } \end{array}\;\right)\right)}{\left(\frac{k_{s2}+\left(m-1\right)k_{bf}-\left(m-1\right)\left(k_{bf}-k_{s2}\right)\phi_{2}}{k_{s2}+k_{bf}\left(m-1\right)+\left(k_{bf}-k_{s2}\right)\phi_{2}}\right)}=0. \end{array}$$

Where, $A^{*}=\frac{V_{0}}{\mathrm{ah}},$ $S=\frac{c}{a},$ $\Omega =\frac{\omega_{0}}{a}$, $\mathrm{Pr}=\frac{\mu_{f}{\left(C_{p}\right)}_{f}}{k_{f}}$. Further, dimensionless flow conditions are attained by plugging the invertible transformations as follows:

(12) $$\begin{array}{c} F\left(\eta \right)=A^{*}\\ \;F^{\prime }\left(\eta \right)=1\\ G\left(\eta \right)=0\\ \beta \left(\eta \right)=1 \end{array}\}at\;\eta =0\;\left(\mathrm{at}\;\mathrm{lower}\;\mathrm{fixed}\;\mathrm{permeable}\;\mathrm{plate}\right),\;$$

And,

(13) $$\begin{array}{c} F\left(\eta \right)=\frac{S}{2}\\ F^{\prime }\left(\eta \right)=0\\ \;G\left(\eta \right)=0\\ \;\beta \left(\eta \right)=0 \end{array}\}at\;\eta =1\;\left(\mathrm{at}\;\mathrm{upper}\;\mathrm{moveable}\;\mathrm{plate}\right).\;$$

### 3.4. Wall Shear Stresses

The analysis of sear stresses is significant. For the particular model, these are defined as:

(14) $$C_{F}=\frac{\Xi_{2}^{*}{\left(\frac{\partial u}{\partial y}\right)}_{y=0}}{\Xi_{1}^{*}U_{0}^{2}},$$

The shear stresses subject to upper plate at y=h(t) described as:

(15) $$C_{F}=\frac{\Xi_{2}^{*}{\left(\frac{\partial u}{\partial y}\right)}_{y=h\left(t\right)}}{\Xi_{1}^{*}U_{0}^{2}},$$

The simplifications of theses expressions yield the following dimensionless version of the shear stresses:

(16) $$Re_{x}C_{F}=\frac{1}{{\left(1-\phi_{1}\right)}^{2.5}{\left(1-\phi_{2}\right)}^{2.5}\left(\left(1-\phi_{2}\right)\left(\left(1-\phi_{1}\right)+\phi_{1}\frac{\rho_{s1}}{\rho_{f}}\right)+\phi_{2}\rho_{s2}\right)}F{}^{\prime \prime }\left(\eta_{=0}\right),$$

And,

(17) $$Re_{x}C_{F}=\frac{1}{{\left(1-\phi_{1}\right)}^{2.5}{\left(1-\phi_{2}\right)}^{2.5}\left(\left(1-\phi_{2}\right)\left(\left(1-\phi_{1}\right)+\phi_{1}\frac{\rho_{s1}}{\rho_{f}}\right)+\phi_{2}\rho_{s2}\right)}F{}^{\prime \prime }\left(\eta_{=1}\right).$$

## 4. Mathematical Analysis

The Variation of Parameters (VPM) technique is adopted to tackled the hybrid model over finite domain. By following the general procedure of VPM, the particular hybrid model takes the following form:

(18) $$\begin{array}{l} F_{n+1}\left(\eta \right)\\ =F\left(0\right)+\eta F^{\prime }\left(0\right)+\frac{\eta^{2}}{2!}F^{\prime \prime }(0)+\frac{\eta^{3}}{3!}F^{\prime \prime \prime }\left(0\right)\\ +\left(\overset{\eta }{\underset{0}{\int }}\frac{{\left(s\eta -s+\left(-\eta s+\eta \right)\right)}^{3}}{3!}\left(\begin{array}{c} \left(\begin{array}{c} \left(\left(1-\phi_{2}\right)\left(\left(1-\phi_{1}\right)+\phi_{1}\frac{\rho_{s1}}{\rho_{f}}\right)+\phi_{2}\rho_{s2}\right)\\ \begin{array}{c} {\left(1-\phi_{1}\right)}^{2.5}{\left(1-\phi_{2}\right)}^{2.5}\left(3F^{\prime }F^{\prime \prime }-FF^{\prime \prime \prime }+\frac{S}{2}\left(3F^{\prime \prime }+\eta F^{\prime \prime \prime }\right)+2\Omega G^{\prime }\right)\\ -{\left(1-\phi_{1}\right)}^{2.5}{\left(1-\phi_{2}\right)}^{2.5} \end{array} \end{array}\right)\\ \left(1+\frac{3\phi \left(\phi_{1}\sigma_{1}+\phi_{2}\sigma_{2}-\sigma_{bf}\left(\phi_{1}+\phi_{2}\right)\right)}{\left(\phi_{1}\sigma_{1}+\phi_{2}\sigma_{2}+2\phi \sigma_{bf}\right)-\sigma_{bf}\phi \left(\left(\phi_{1}\sigma_{1}+\phi_{2}\sigma_{2}\right)-\sigma_{bf}\left(\phi_{1}+\phi_{2}\right)\right)}\right)M^{2}F^{\prime \prime } \end{array}\right)ds\right) \end{array}$$

(19) $$\begin{array}{l} G_{n+1}\left(\eta \right)\\ =G\left(0\right)+\eta G^{\prime }\left(0\right)\\ -\overset{\eta }{\underset{0}{\int }}\frac{\left(\eta^{2}s-s+\left(\eta -s\eta^{2}\right)\right)}{1!}\left(\begin{array}{c} \left(\left(1-\phi_{2}\right)\left(\left(1-\phi_{1}\right)+\phi_{1}\frac{\rho_{s1}}{\rho_{f}}\right)+\phi_{2}\rho_{s2}\right)\\ \begin{array}{c} {\left(1-\phi_{1}\right)}^{2.5}{\left(1-\phi_{2}\right)}^{2.5}\left(FG^{\prime }-F^{\prime }G+2\Omega F^{\prime }-S\left(G+\frac{\eta }{2}G^{\prime }\right)\right)\\ -{\left(1-\phi_{1}\right)}^{2.5}{\left(1-\phi_{2}\right)}^{2.5} \end{array}\\ \left(1+\frac{3\phi \left(\phi_{1}\sigma_{1}+\phi_{2}\sigma_{2}-\sigma_{bf}\left(\phi_{1}+\phi_{2}\right)\right)}{\left(\phi_{1}\sigma_{1}+\phi_{2}\sigma_{2}+2\phi \sigma_{bf}\right)-\sigma_{bf}\phi \left(\left(\phi_{1}\sigma_{1}+\phi_{2}\sigma_{2}\right)-\sigma_{bf}\left(\phi_{1}+\phi_{2}\right)\right)}\right)M^{2}G \end{array}\right)ds \end{array}$$

(20) $$\begin{array}{l} \beta_{n+1}\left(\eta \right)\\ =\beta \left(0\right)+\eta \beta^{\prime }\left(0\right)\\ +\overset{\eta }{\underset{0}{\int }}\frac{\left(\eta^{2}s-s+\left(\eta -s\eta^{2}\right)\right)}{1!}\left(\begin{array}{c} \left(\frac{1}{\left(1+\frac{Rd}{\left(\frac{k_{s2}+\left(m-1\right)k_{bf}-\left(m-1\right)\left(k_{bf}-k_{s2}\right)\phi_{2}}{k_{s2}+k_{bf}\left(m-1\right)+\left(k_{bf}-k_{s2}\right)\phi_{2}}\right)}\right)}\right)\\ \frac{\mathrm{Pr}\left(\left(1-\phi_{2}\right)\left(\left(1-\phi_{1}\right)+\phi_{1}\frac{{\left(\rho c_{p}\right)}_{s1}}{{\left(\rho c_{p}\right)}_{f}}\right)+\phi_{2}{\left(\rho c_{p}\right)}_{s2}\right)}{\left(\frac{k_{s2}+\left(m-1\right)k_{bf}-\left(m-1\right)\left(k_{bf}-k_{s2}\right)\phi_{2}}{k_{s2}+k_{bf}\left(m-1\right)+\left(k_{bf}-k_{s2}\right)\phi_{2}}\right)}\\ \begin{array}{c} \left(\begin{array}{c} F\beta^{\prime }\\ -\frac{S}{2}\eta \beta^{\prime } \end{array}+\gamma \left(\frac{F^{\prime }\beta^{\prime }S\eta }{2}-\frac{3S^{2}\eta \beta^{\prime }}{4}-\frac{S^{2}\eta^{2}\beta^{\prime \prime }}{4}+S\eta \beta^{\prime \prime }+\frac{3SF\beta^{\prime }}{2}\begin{array}{c} --FF^{\prime \beta^{\prime }}\\ -F^{2}\beta^{\prime \prime } \end{array}\;\right)\right) \end{array} \end{array}\right)ds \end{array}$$

Now, consuming the initial conditions and adjust $F^{\prime \prime }\left(0\right)=\alpha_{1}^{*},\;F^{\prime \prime \prime }\left(0\right)=\alpha_{2}^{*},\;G^{\prime }\left(0\right)=\alpha_{3}^{*}$ and $\beta^{\prime }\left(0\right)=\alpha_{4}^{*}$ and assigned the following initial guesses for the model:

(21) $$\;F_{0}\left(\eta \right)=A+\eta +\frac{\eta^{2}}{2!}\alpha_{1}^{*}+\frac{\eta^{3}}{3!}\alpha_{2}^{*}$$

(22) $$\;G_{0}\left(\eta \right)=\eta \alpha_{3}^{*}$$

(23) $$\;\beta_{0}\left(\eta \right)=1+\eta \alpha_{4}^{*}$$

After plugging these initial trials in Equations (16)–(18), higher order approximations of the solutions obtained.

## 5. Conclusions

Heat transfer remains a major problem of the industrialist to accomplish the processing of various industrial products. After the development of nanofluids and a premier class of fluids called hybrid nanofluids, these problems reduced, and researchers are studying the heat transfer characteristics under various physical situations. Thus, a novel thermal transport analysis by incorporating the effects of thermal radiations for hybrid nanofluid is presented and found significant results for the heat transfer. From the presented analysis, it is perceived that:

The velocity of GO-MoS_2_/H_2_O-C_2_H_6_O_2_ promptly rises for stronger squeezing effects in the presence of constant suction at the lower plate.

The rotational velocity of GO-MoS_2_/H_2_O-C_2_H_6_O_2_ increases for a higher magnetic parameter and the back flow is examined in the locality of the middle portion of the channel.

The temperature β(η) rises in the presence of thermal relaxation parameter for constant suction and almost inconsequential variations in the temperature are examined for thermal radiation.

The rapid increasing trends in the shear stresses are observed at the upper plate.

The thermal conductivity of GO-MoS_2_/H_2_O-C_2_H_6_O_2_ prevailed, and is better suited for industrial and engineering uses.

In future, the model can be modified for various hybrid nanofluids under various physical conditions.

## Figures and Tables

**Table 1:** Comparative analysis of current results with existing scientific study, $\phi_{1}=\phi_{2}=0.$

Ω.	A*	S	Present Results		[21]	
			$F{}^{\prime \prime }\left(0\right)$	$F{}^{\prime \prime }\left(1\right)$	$F{}^{\prime \prime }\left(0\right)$	$F{}^{\prime \prime }\left(1\right)$
**2**	**0.5**	−1	−10.5312	7.70317	−10.5311	7.7031
		0	−7.58901	4.82359	−7.5890	4.8235
		1	−4.51255	1.80917	−4.5125	1.8091
		2	−1.28942	−1.35423	−1.2894	−1.3542
		3	2.0835	−4.67005	2.0835	−4.67005
	0.0	2	2.00374	−4.58271	2.0037	−4.5827
	0.3		0.06127	−2.62279	0.06127	−2.62278
	0.6		−1.9823	−0.731299	−1.9823	−0.731299
	0.9		−4.1349	1.09241	−4.1349	1.09241
	1.2		−6.4047	2.84917	−6.4047	2.84916

**Table 2:** Effective models for conventional and hybrid nanofluids.

$\Xi_{i}^{*}$	Properties	$Graphene\;Oxide/C_{2}H_{6}O_{2}-H_{2}O\;\mathrm{Regular}\;\mathrm{Nanofluid}\;$	$GO-Molybdenum\;disulfide/C_{2}H_{6}O_{2}-H_{2}O$ 50:50 Hybrid Nanofluid
$\Xi_{1}^{*}$	Density	$\frac{\rho_{nf}}{\left(\left(1-\phi \right)+\phi \frac{\rho_{s}}{\rho_{f}}\right)\;}=\rho_{f}$	$\frac{\rho h_{nf}}{\left(\left(1-\phi_{2}\right)\left(\left(1-\phi_{1}\right)+\phi_{1}\frac{\rho_{s1}}{\rho_{f}}\right)+\phi_{2}\rho_{s2}\right)\;}=\rho_{f}$
$\Xi_{2}^{*}$	Dynamic viscosity	$\mu_{nf}{\left(1-\phi \right)}^{2.5}=\mu_{f}$	$\mu_{hnf}{\left(1-\phi_{1}\right)}^{2.5}{\left(1-\phi_{2}\right)}^{2.5}=\mu_{f}$
$\Xi_{3}^{*}$	Electrical conductivity	$\frac{\sigma_{nf}}{\left(1+\frac{\left(\frac{\sigma_{s}}{\sigma_{f}}-1\right)\phi }{\left(\frac{\sigma_{s}}{\sigma_{f}}+2\right)-\left(\frac{\sigma_{s}}{\sigma_{f}}-1\right)\phi }\right)}=\sigma_{f}$	$\frac{\sigma_{hnf}}{\left(1+\frac{3\phi \left(\phi_{1}\sigma_{1}+\phi_{2}\sigma_{2}-\sigma_{bf}\left(\phi_{1}+\phi_{2}\right)\right)}{\left(\phi_{1}\sigma_{1}+\phi_{2}\sigma_{2}+2\phi \sigma_{bf}\right)-\sigma_{bf}\phi \left(\left(\phi_{1}\sigma_{1}+\phi_{2}\sigma_{2}\right)-\sigma_{bf}\left(\phi_{1}+\phi_{2}\right)\right)}\right)}=\sigma_{bf}$
$\Xi_{4}^{*}$	Thermal conductivity	$\frac{k_{nf}}{\left(\frac{k_{s}+\left(m-1\right)k_{f}-\left(m-1\right)\left(k_{f}-k_{s}\right)\phi }{k_{s}+\left(m-1\right)k_{f}+\left(k_{f}-k_{s}\right)\phi }\right)}=k_{f}$	$\begin{array}{c} \frac{k_{hnf}}{\left(\frac{k_{s2}+\left(m-1\right)k_{bf}-\left(m-1\right)\left(k_{bf}-k_{s2}\right)\phi_{2}}{k_{s2}+k_{bf}\left(m-1\right)+\left(k_{bf}-k_{s2}\right)\phi_{2}}\right)=k_{bf}\;and}\\ \frac{k_{bf}}{\left(\frac{k_{s1}+\left(m-1\right)k_{f}-\left(m-1\right)\left(k_{f}-k_{s1}\right)\phi_{1}}{k_{s1}+\left(m-1\right)k_{f}+\left(k_{f}-k_{s1}\right)\phi_{1}}\right)}=k_{f}\; \end{array}$
$\Xi_{5}^{*}$	Heat capacity	$\frac{{\left(\rho c_{p}\right)}_{nf}}{\left(1-\phi +\frac{\phi {\left(\rho c_{p}\right)}_{s}}{{\left(\rho c_{p}\right)}_{f}}\right)}={\left(\rho c_{p}\right)}_{f}$	$\frac{{\left(\rho c_{p}\right)}_{hnf}}{\left(\left(1-\phi_{2}\right)\left(\left(1-\phi_{1}\right)+\phi_{1}\frac{{\left(\rho c_{p}\right)}_{s1}}{{\left(\rho c_{p}\right)}_{f}}\right)+\phi_{2}{\left(\rho c_{p}\right)}_{s2}\right)\;}={\left(\rho c_{p}\right)}_{s}$
$\Xi_{6}^{*}$	Thermal expansion coefficient	$\frac{{\left(\rho \beta \right)}_{nf}}{\left(1-\phi +\frac{\phi {\left(\rho \beta \right)}_{s}}{{\left(\rho \beta \right)}_{f}}\right)}={\left(\rho \beta \right)}_{f}$	$\frac{\left(\rho \beta \right)h_{nf}}{\left(\left(1-\phi_{2}\right)\left(\left(1-\phi_{1}\right)+\phi_{1}\frac{{\left(\rho \beta \right)}_{s1}}{{\left(\rho \beta \right)}_{f}}\right)+\phi_{2}{\left(\rho \beta \right)}_{s2}\right)\;}$

**Table 3:** Different shapes of tiny particles.

Bricks	Cylinders	Platelets
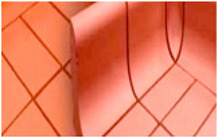	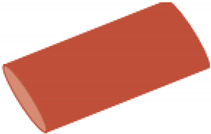	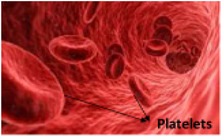
Shape Factor for Bricks	Shape Factor for Cylinders	Shape Factor for Platelets
m=3.7	m=4.9	m=5.7

**Table 4:** The values of the thermophysical characteristics.

Thermophysical Characteristics	Unit	Hybrid Fluid Phase	Nanomaterials	
		$C_{2}H_{6}O_{2}-H_{2}O$	$\mathrm{Molybdenum}\;\mathrm{Disulfide}\;({\mathrm{MoS}}_{2})$	Graphene Oxide (GO)
ρ	$\mathrm{kg}/m^{3}$	1063.8	5060	1800
$c_{p}$	J/kgK	3630	397.21	717
k	W/mK	0.387	904.4	5000
$\beta^{*}$	1/K	$5.8\times 10^{-4}$	$2.8424\times 10^{-5}$	$2.84\times 10^{-4}$
σ	1/Ωm	$9.75\times 10^{-4}$	$2.09\times 10^{4}$	$6.30\times 10^{7}$

## Nomenclature

GO	Graphene oxide
MoS_2_	Molybdenum disulfide
Φ	volume fraction
$\rho_{\mathrm{nf}}$	nanofluids density
$\mu_{\mathrm{nf}}$	effective dynamic viscosity
${\left({\rho c}_{p}\right)}_{\mathrm{nf}}$	effective heat capacity
nf	stands for nanofluid
hnf	stands for hybrid nanofluid
$\phi_{1}$	volume fraction of GO nanoparticles
$\phi_{2}$	volume fraction of MoS_2_ nanoparticles
$\sigma_{1}$	electrical conductivity of GO
$\sigma_{2}$	electrical conductivity of MoS_2_
$k_{\mathrm{nf}}$	thermal conductivity of nanofluid
$k_{\mathrm{bf}}$	thermal conductivity of base liquid
$k_{\mathrm{hnf}}$	thermal conductivity of hybrid nanofluid
u, v, w	velocities along x, y and z directions, respectively
T	temperature
η	dimensionless variable
F(η)	dimensionless velocity
G(η)	rotational velocity
β(η)	dimensionless temperature
A^*^	suction parameter
S	squeezed parameter
Rd	thermal radiation parameter
γ	thermal relaxation parameter
Pr	Prandtl number
